# Recent advances in experimental techniques to probe fast excited-state dynamics in biological molecules in the gas phase: dynamics in nucleotides, amino acids and beyond

**DOI:** 10.1098/rspa.2013.0458

**Published:** 2013-11-08

**Authors:** Michael Staniforth, Vasilios G. Stavros

**Affiliations:** Department of Chemistry, University of Warwick, Library Road, Coventry CV4 7AL, UK

**Keywords:** spectroscopy, ultrafast, dynamics

## Abstract

In many chemical reactions, an activation barrier must be overcome before a chemical transformation can occur. As such, understanding the behaviour of molecules in energetically excited states is critical to understanding the chemical changes that these molecules undergo. Among the most prominent reactions for mankind to understand are chemical changes that occur in our own biological molecules. A notable example is the focus towards understanding the interaction of DNA with ultraviolet radiation and the subsequent chemical changes. However, the interaction of radiation with large biological structures is highly complex, and thus the photochemistry of these systems as a whole is poorly understood. Studying the gas-phase spectroscopy and ultrafast dynamics of the building blocks of these more complex biomolecules offers the tantalizing prospect of providing a scientifically intuitive *bottom-up* approach, beginning with the study of the subunits of large polymeric biomolecules and monitoring the evolution in photochemistry as the complexity of the molecules is increased. While highly attractive, one of the main challenges of this approach is in transferring large, and in many cases, thermally labile molecules into vacuum. This review discusses the recent advances in cutting-edge experimental methodologies, emerging as excellent candidates for progressing this *bottom-up* approach.

## 1. Introduction

**Figure 1.**
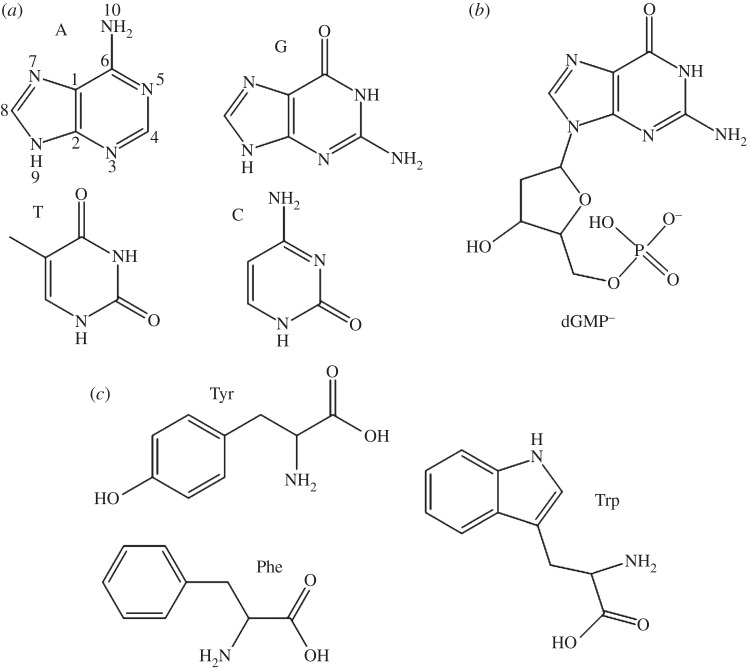
(*a*) The DNA nucleobases adenine (A), guanine (G), cytosine (C), thymine (T), (*b*) the nucleotide anion 2′-deoxy-guanosine 5′-monophosphate^−^ (dGMP^−^) and (*c*) aromatic amino acids tyrosine (Tyr), phenylalanine (Phe) and tryptophan (Trp).

Chemical reactions, at their most basic level, can be described as the interaction between electrons of different atoms and molecules. As a result, understanding the properties of the electronic states that these electrons occupy is vitally important towards understanding a vast array of chemistry and biology, such as the complex transformations taking place in naturally occurring systems involved in photosynthesis ([1–5] and references therein), metabolic processes [6], nucleoside hydrolase reactions [7] and so on. In many chemical reactions, there is an initial energy barrier, the activation barrier that must be overcome for that reaction to proceed, and so it is often the case that chemistry must take place with one or more of the reactants in some energetically excited state, particularly in light-driven processes, i.e. photochemistry. For the purposes of this review, we consider the photochemistry of the nucleobases, nucleotides and aromatic amino acids, including derivatives thereof, following irradiation with ultraviolet (UV) light; some of these molecules under discussion are depicted in figure 1. In particular, we focus on the recent advances in cutting-edge gas-phase experimental methodologies that have made this possible.

Photoexcitation of molecules with UV radiation populates electronically excited states, the dynamics of which are highly varied and depend on a wide range of processes—such as fluorescence, internal conversion (IC) and photodissociation, to name but a few—which are often in kinetic competition. Therefore, understanding the underlying dynamics of these competing processes is critical towards understanding light-activated chemistry. For instance, upon irradiation with UV light, the purine-derived nucleobases (adenine and guanine) and pyrimidine-derived nucleobases (cytosine and thymine; figure 1*a*) become electronically excited, leading to a finite probability that two adjacent nucleobases on one of the helical backbones of DNA will covalently bond to each other, potentially leading to mutagenic miscoding of the DNA sequence or even lethal noncoding [8,9]. Common photodissociation by-products are cyclobutane-pyrimidine dimers, or 6,4-photoproduct pyrimidine adducts [10,11].

In large biological systems, our understanding of these excited-state dynamics is considerably hampered owing to the vast and varied array of processes that can occur, not only within individual subunits of the polymeric biomolecule, but between subunits themselves or with the surrounding solvent. This degree of complexity has led to efforts aimed at simplifying the problem through the study of individual subunits in an isolated environment, i.e. isolated UV chromophores in the gas phase. Such studies have many advantages. As well as the greatly simplified chemical landscape presented by smaller molecules, gas-phase experiments allow for far more differential measurements than the solution phase, permitting studies resolved in time, kinetic energy, pump/probe photon energy, mass, etc. Investigating the excited-state dynamics of individual subunits using such a *bottom-up* approach has had considerable success in elucidating some of the key decay mechanisms at work in these molecular subunits. For example, the nucleobases are known to significantly absorb radiation in the UV region of the electromagnetic spectrum, predominantly resulting in a 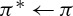 transition; yet, the quantum yield of fluorescence of these molecules following such absorption is relatively low (approx. 10^−4^) above certain excitation energy thresholds [8,9]. This suggests that there are relaxation mechanisms operating within these molecules to reduce the ^1^ππ* state lifetime. Not only does this result in a significant reduction in the fluorescence quantum yield, but there is also a reduced probability of undesired excited-state photoreactions [8].

**Figure 2.**
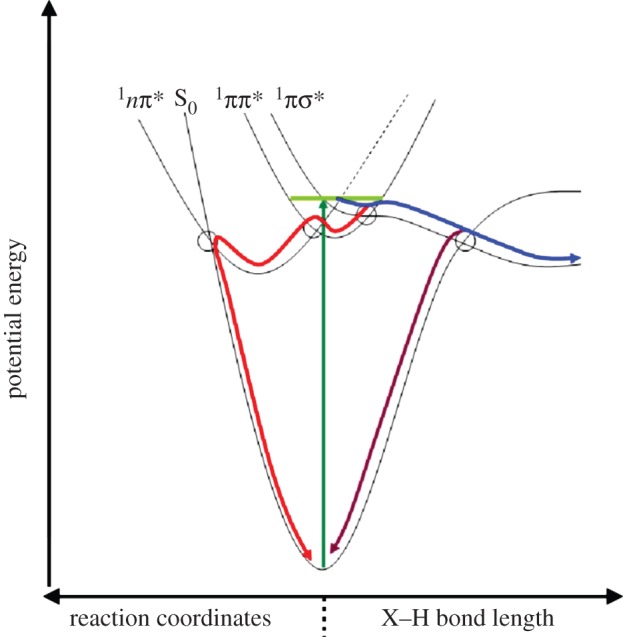
Schematic potential energy surfaces and major relaxation pathways in biologically relevant molecules following excitation to the 1ππ* state (light green line). One of the dominant pathways in DNA bases is 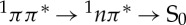 (red) along ring-distortion coordinates. Evidence also exists for a 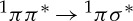 pathway leading either to H-atom elimination (blue) or IC down to S_0_ (purple) along X−H bonds where X=O or N. CIs mediating non-radiative pathways are circled where appropriate (adapted from [18–20]).

A great deal of work has already been carried out on exploring the excited-state dynamics of the nucleobases and aromatic amino acid chromophores in the gas phase, and the reader is referred to [12–15] for further details. Only a brief discussion of this work is presented here as a means of setting the scene for the remainder of this review. The excited-state dynamics of nucleobases have been studied using time-resolved mass spectroscopy (TR-MS), time-resolved photoelectron spectroscopy (TR-PES) and time-resolved velocity map ion imaging (TR-VMI), revealing femtosecond (fs)–picosecond (ps) long relaxation dynamics ensuing from the initially populated ^1^ππ* state. Adenine has been the most widely studied of the nucleobases, generating conflicting conclusions pertaining to the deactivation pathways; for example, the discussion as to whether sequential IC from 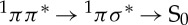 is in kinetic competition with sequential IC from 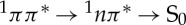 following 267 nm photoexcitation [16,17], both of which are mediated through appropriate conical intersections (CIs) illustrated in figure 2 (blue and red lines, respectively; it should be noted here that other pathways have been suggested—see review [21]). As with the nucleobases, TR-MS, TR-PES and TR-VMI have also been successfully implemented to study a range of aromatic amino acid chromophores (e.g. the indole moiety of tryptophan, see figure 1*c*). An additional deactivation mechanism worthy of note is tunnelling from the 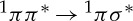 (not shown in figure 2), beneath the ^1^ππ*/^1^πσ* CI, as proposed to explain the nanosecond-long dynamics observed in phenol (the chromophore of tyrosine) [19,22–25].

The focus of this review is to introduce the reader to the most recent advances in extending these gas-phase studies to larger biomolecules, for instance, polymeric subunits. However, before we discuss progress in this burgeoning field, it is important to provide a brief background to the existing experimental methodologies (TR-MS, TR-PES and TR-VMI) implemented to study nucleobases and aromatic amino acid chromophores (§2). We will then progress to discuss the current ‘state of the art’ in investigating excited-state dynamics of larger biomolecules, focusing on nucleobases and derivatives of nucleobases using laser desorption (LD) (§3*a*), amino acids with laser-induced acoustic desorption (LIAD) (§3*b*) and finally charged nucleotides, amino acid precursors and dipeptides using electrospray ionization (§3*c*). The review will present some concluding remarks and a brief discussion on the future outlook (§4).

## 2. Experimental methodologies using molecular beam methods

As has been briefly alluded to, the majority of excited-state dynamics measurements on nucleobases in the gas phase have been carried out on adenine and its derivatives. The reason for this is twofold: (i) adenine is stable upon heating (thus presenting less of a challenge to transfer it into vacuum) and (ii) adenine has only one dominant stable tautomer [12], making the analysis of the experimental results much easier to interpret. This is in stark contrast to guanine, which has multiple conformers and undergoes thermal decomposition upon heating [26]. While there is a wealth of knowledge emerging in the scientific literature on the excited-state dynamics of nucleobases and aromatic amino acid chromophores (presented in the individual subsections below), it is unclear whether the photochemistry of these subunits translates to larger systems such as nucleotides, polynucleotides and polypeptides. The gas-phase study of these larger systems presents certain challenges, which will be better understood after considering the limitations of the current standard in gas-phase experimental techniques. This section serves to demonstrate the currently implemented (TR-MS, TR-VMI and TR-PES) methodologies used to study excited-state dynamics of nucleobases and aromatic amino acid chromophores.

### (a) Time-resolved mass spectrometry

**Figure 3.**
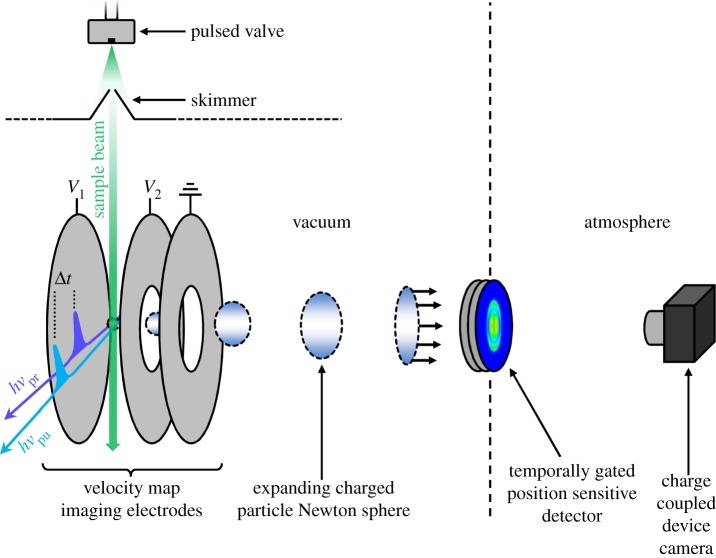
Schematic of a velocity map imaging arrangement used to perform TR-MS, TR-VMI or TR-PES experiments. *V*_1_ and *V*_2_ correspond to the voltages applied to the repeller and accelerator electrodes, respectively. For VMI, the voltage ratio *V*_1_:*V*_2_ is set to approximately 1:0.7. Further information regarding each technique is provided in §2.

TR-MS uses time-of-flight mass spectrometers (TOF-MSs). In these experiments (and also TR-VMI and TR-PES), a skimmed molecular beam of target molecules is produced by seeding a vapour pressure of the molecule of interest into an inert carrier gas such as Ar or He (typically a few bars), introduced into vacuum using a pulsed valve [27]. Such molecular beams have the advantage of producing high sample densities of translationally cold molecules (the majority of molecules will be in their vibrationless ground state) allowing state-selective experiments and the possibility of a reduced number of conformers present [28]. The seeded molecular beam pulse is intercepted perpendicularly by fs pump (to prepare the excited state of the analyte) and probe (to ionize the excited molecules) laser pulses at the centre of the TOF electrostatic optics, usually replicating the arrangement described by Wiley & McLaren [29], a modified version of which is schematically shown in figure 3. A mass spectrum of (positive) ions, resolved by their different flight times, is then collected by a detector placed at the terminus of a flight tube. By recording a series of mass spectra at various time delays (Δ*t*) between pump and probe pulses, one is able to track the excited-state dynamics of both the parent molecule and subsequent fragments by monitoring the associated ions. This approach has been successfully used to study excited-state dynamics in nucleobases [18,30,31], phenol [22,32], indole [33], pyrrole [34–36] and imidazole [37,38].

### (b) Time-resolved velocity map ion imaging

VMI, along with its time-resolved counterpart, TR-VMI, has revolutionized the field of photofragment spectroscopy and is now one of the essential methodologies used in gas-phase molecular reaction dynamics ([39,40] and references therein). One of the major advantages of VMI is that it simultaneously recovers both the recoil speed (and thus kinetic energies) and the angular recoil trajectories (velocity vectors) of the original three-dimensional distribution of ionized photofragments (Newton sphere) by collecting its two-dimensional projection. Such angular distribution measurements grant the researcher a wealth of useful information regarding the processes involved in photodissociation: recoil directionality, molecular alignment as a function of time, scattering, etc. [39,41,42]. With reference to figure 3, VMI is typically implemented using a *gridless* Wiley–McLaren TOF electrode arrangement [43], which both temporally and spatially focuses ions onto a position sensitive detector, usually a pair of microchannel plates coupled to a phosphor screen placed at the terminus of a field-free flight tube. By temporally gating the detector, it is possible to collect the two-dimensional projection of only a specific photofragment mass of interest, based on its known TOF. As with TR-MS, ion images are collected at a series of pump–probe time delays from which one is able to track the dynamics of fragment ions.

Unlike the gridded electrodes used in a standard Wiley–McLaren TOF-MS, the gridless electrodes used in VMI lead to the formation of an electrostatic lens, which causes ionized photofragments with the same velocity vectors to be focused onto the same position of the two-dimensional detector plane, regardless of their initial position within the ionization volume of the focused probe laser. As a result, carefully designed VMI arrangements can deliver energy resolution down to Δ*E*/*E*=0.38% [44]. Although this energy resolution is not achievable in ultrafast TR-VMI experiments, owing to the inherently broad spectral bandwidth of fs pulses (typically hundreds of cm^−1^), coupling ultrafast pump–probe spectroscopy with VMI still affords temporal *and* energy information. In addition, TR-VMI vitally affords photofragment angular recoil information regarding the photodissociation mechanisms. By combining the time, energy and angular recoil information, one is able to glean detailed insights into the excited-state dynamics that precede photodissociation. TR-VMI has been successfully used to study excited-state dynamics in adenine [17], phenol [19,45], indole [46], pyrrole [36] and imidazole [47].

### (c) Time-resolved photoelectron spectroscopy

TR-PES is one of the most versatile and highly differential methodologies used to probe ultrafast chemical reaction dynamics in the gas phase [14,48–51]. In particular, it is able to monitor dynamics flowing between both optically ‘bright’ states (^1^ππ*) and optically ‘dark’ (typically ^1^nπ* and ^1^πσ*) states [12,52] and, in principle, is able to return a complete dynamical map along the entire reaction coordinate provided the ionizing probe photon has enough energy to detach an electron from any location along the reaction coordinate ([53] and references therein). With regards to experimental approaches for performing TR-PES, these have been comprehensively reviewed in the current literature [54–56]. The most commonly implemented approaches use either a magnetic-bottle analyser [57] or a velocity map photoelectron imaging arrangement [43,58], similar to that shown in figure 3; note that only the latter is able to return additional information regarding photoelectron angular distributions [41].

After photoexcitation of the molecule of interest with an fs pump pulse, the excited-state population evolves along the reaction coordinate, over time, Δ*t*. A time-delayed fs probe pulse then projects the evolving system onto a final state, the molecular cation, together with a correlated photoelectron. The kinetic energy signatures of the detected electrons can change in both energy and intensity, according to the time-dependent ionization cross sections, dictated by the Franck–Condon overlap between the excited neutral molecule and the vibronic levels in the corresponding cation. This provides a blueprint for untangling the ultrafast excited-state dynamics. In a simplified Koopmans' picture, at a given Δ*t*, the electron kinetic energy is given by the difference between the sum of the pump and probe photon energies (*hν*_pu_+*hν*_pr_) and the sum of the adiabatic ionization energy and vibrational energy of the final cation state (IE+*E*_f_) [59], thus
2.1
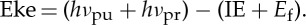


## 3. Excited-state dynamics: the current state of the art

One major hurdle to overcome in all of these techniques, and one that has significant implications on the work being considered in this review, is that they generally require fairly high sample densities (typically not less than 10^12^ molecules cm^−3^) [60,61] to produce statistically significant results. In many multi-photon processes, the absolute number of ions detected is relatively small (sometimes less than one count per laser shot [62,63]). As such, it is important to seed the molecular beam with a large number of sample molecules. In the case of the smaller molecules discussed earlier, which are relatively volatile and sufficiently stable under heating, this is a simple task of passing an inert carrier gas (typically argon or helium) over a solid or bubbling it through a liquid sample. This is not possible, however, when studying more thermally labile biologically relevant molecules, for example, nucleotides, which are more prone to thermal decomposition and fragmentation when heated [64]. The challenge then, is to find some alternative method of transferring a sufficiently large number of sample molecules into the gas phase to perform spectroscopy and bypassing decomposition or fragmentation of the target molecules.

The remainder of this section will present the most cutting-edge techniques employed in addressing this problem. Although many of the following techniques are well established within the mass spectrometry community, their adaptation as sources of generating isolated molecules, be they neutrals or ions, to perform ultrafast gas-phase spectroscopy has been far more recent. We present an overview of the very latest experimental results obtained through application of these techniques, demonstrating the significant potential that these techniques offer towards excited-state dynamics of much larger biological molecules in vacuum.

### (a) Excited-state dynamics using laser desorption

The principles behind LD have long been understood. Experiments using a technique perceived as LD were performed as early as 1968 by Vastola & Pirone [65], in which a ruby laser was used to irradiate organic samples, resulting in the production of ions. However, it was not until a decade later that Posthumus *et al.* [66] demonstrated that the technique could be used to produce mass spectra of not just organic salts, but also other polar, non-volatile biochemicals, including nucleotides [67]. In these early experiments [65,66], the sample, dissolved in a solvent, was deposited onto a metal. Once the solvent was evaporated, it left a layer of the sample molecules loosely bound to the metal surface. Ionization was a direct result of the desorption process.

Later developments of this technique enabled scientists to study the spectroscopy of neutral molecules; these molecules were first desorbed by a laser pulse, and then ionized by a second laser pulse. Notable examples involving biomolecules included the electronic spectra of tryptophan peptides [68], resonance enhanced multi-photon ionization (REMPI) spectroscopy of tryptophan [69], REMPI of dipeptides [70], double resonance laser spectroscopy of dipeptides [71] and the photochemistry of purine nucleobases [72]. While LD has been widely used to study the spectroscopy of a number of biomolecules, the LD variant, matrix-assisted laser desorption ionization [73–75], has found widespread use in the mass spectrometry community. However, despite there being no reason why the technique could not be applied for spectroscopic studies, few examples exist in the literature, and as such we do not discuss this approach any further in this review.

A major point of consideration for LD techniques is the inherent instability of the gaseous sample produced. It is a very difficult task to uniformly distribute the sample molecule around a matrix or metal substrate, and as such, it is necessary to perform significant signal averaging to produce consistent results from such experiments ([76] and references therein). This aspect presents a particular problem for fs time-resolved dynamics studies; it is essential to keep the ionization events per laser pulse relatively low to prevent the high field intensities of the fs pulses causing unwanted nonlinear effects (e.g. ac Stark shifts [77], multi-photon processes, etc.). In experiments such as photoelectron/photoion coincidence [78], the event counts must be fewer than a single count per laser shot. At rates of 10 Hz, this would require taking measurements over several days to achieve similar levels of ion signal seen in more well-established sources used in molecular beam experiments (see §2).

**Figure 4.**
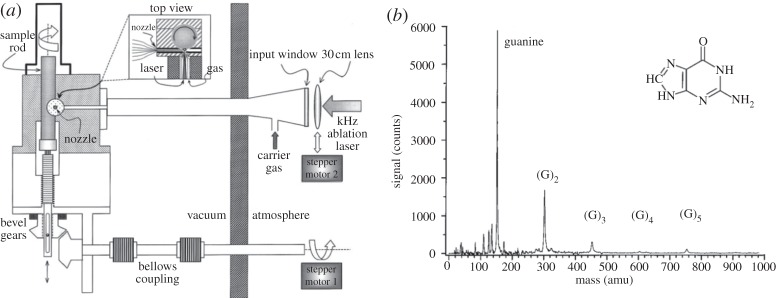
(*a*) kHz LD experimental set-up and (*b*) mass spectrum of guanine obtained via a kHz LD experiment. Reproduced with permission from [62].

It is highly beneficial (although not essential) in ultrafast experiments to be able to acquire ion signals at rates of 1 kHz (or greater), not only for signal averaging, but also given that this is the repetition rate of most commercial fs lasers. Experiments designed with exceptional long-lived stability as the focus (which is necessary for high repetition rates) are sparse [62,79]. However, recent experiments by Smits *et al.* [62] have shown that it is possible to produce a viable kHz repetition rate LD source, dramatically reducing the time required to carry out such measurements. In their experiments, it was successfully demonstrated that guanine, compressed into a cylindrical rod, approximately 6 mm in diameter and up to 3 cm in length, could be laser desorbed using an Nd : YLF laser outputting greater than a 10 mJ pulse^−1^ in 150 ns at 527 nm. This desorption source is depicted in figure 4*a*. The cylindrical rod was translated and rotated simultaneously via a screw thread coupled with a stepper motor. The motion of the rod was such that a fresh part of the rod was available for desorption at the repetition rate of the laser (i.e. every 1 ms). In addition to this motion, a second motor was used to dither the position of the focusing lens of the desorption laser, in an asynchronous fashion. This essentially randomized the exact path of the laser over the rod. At kHz repetition rates, Smits *et al.* found a nozzle diameter of no less than 2 mm was necessary to allow for long-term stability. Under these conditions, it was possible to run experiments for upwards of 12 h. Figure 4*b* depicts a TOF-MS of seeded guanine, resonantly ionized following 277 nm radiation with a 150 fs laser pulse, at a count rate of 0.3 counts per laser shot. The mass spectrum shows cluster formation of guanines, illustrating a cold molecular beam expansion. This clustering is likely facilitated by the length of channel between the sample rod and nozzle through which both the sample and carrier gas must pass through, enhancing collisions between molecules, and thus causing clustering in the cold molecular beam. A similar process could also be used to allow for microsolvation of the desorbed molecules with a solvent seeded into the carrier gas. As such, the successful demonstration of a kHz LD source shows the exciting prospect the future holds for time-resolved pump–probe measurements to be coupled to such a source. This will enable dynamics measurements to be carried out on both isolated biomolecules and potentially solvated biomolecules, the latter of which will go some way towards replicating the *natural* environments in which these biomolecules exist.

While a kHz experiment is advantageous for signal averaging, it is by no means essential. There are many successful examples of LD being used to perform spectroscopy on DNA subunits [79–82]. However, there are very few examples that combine LD with pump–probe spectroscopy and, to the best of our knowledge, none that combine LD with ultrafast pump–probe spectroscopy. For example, Gengeliczki *et al.* have successfully used a 10 Hz repetition rate source to study the excited-state dynamics of modified nucleobases, such as 2,4-diaminopyrimidine (2,4-DAPy) and 2,6-diaminopurine (2,6-DAPu) [83] as well as the RNA base uracil [84]. For the purposes of this review, we choose to focus on the modified nucleobases. In this work, a sample was mounted onto a flat, translating stage, approximately 1 mm in front of the nozzle of a jet expansion of 6 atm of argon from a pulsed solenoid valve. The side of the stage facing away from the nozzle was chamfered to prevent any disturbance of the gas expansion. A desorption laser was then aimed onto the sample, 2 mm in front of the nozzle. The fundamental of an Nd : YAG laser (1064 nm) was used, attenuated to 1 mJ cm^−2^ and focused to a diameter of 0.5 mm. The sample/carrier gas mixture was expanded through a rectangular slit nozzle (1×4 mm) into a Wiley–McLaren TOF-MS [85]. The excited-state dynamics of both 2,4-DAPy and 2,6-DAPu following excitation of the 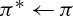 origin band were then probed with time-delayed 266 nm radiation using resonant two-photon ionization (R2PI).

**Figure 5.**
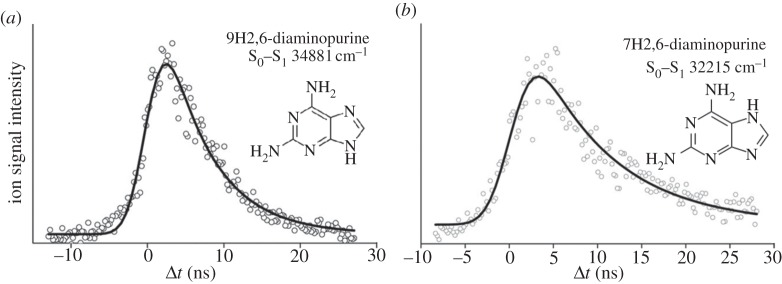
Excited-state lifetime measurements of 2,6-diaminopurine (*a*) 9H and (*b*) 7H tautomers. Reproduced with permission from [83].

For 2,6-DAPu, two tautomers were detected in the molecular beam, the 9H and 7H tautomers (inset, figure 5). The excited-state dynamics following excitation at the origin bands of each of these tautomers is shown in figure 5, leading to time constants for the decay of the initially populated ^1^ππ* state (S_1_) of 6.3±0.4 ns and 8.7±0.8 ns for the 9H (figure 5*a*) and 7H conformers (figure 5*b*), respectively. For 2,4-DAPy, only one tautomer was observed, with an excited-state lifetime between 10 ps and 1 ns. The upper limit is based on the time resolution of the instrument (approx. 1 ns), whereas the lower limit was established based on the sharpness of the peaks observed in the R2PI spectrum. While these values appear to be significantly longer than the time required to produce damaging mutations in DNA, and hence are counterintuitive to the photostability arguments presented in the introduction, the measured lifetimes are significantly longer than the nucleobases themselves, in particular, the purine nucleobases that 2,4-DAPy and 2,6-DAPu mimic [18]. However, these experiments were carried out in a narrow excitation window, very close to the origin band where the lifetimes are expected to be the longest [18,30].

### (b) Excited-state dynamics using laser-induced acoustic desorption

**Figure 6.**
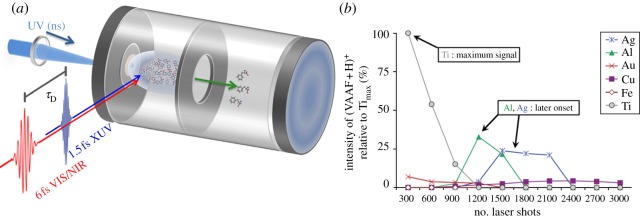
(*a*) Diagram of a typical LIAD source. Reproduced with permission from [87]. (*b*) LIAD signal intensity for various metal foil substrates. Reproduced with permission from [88].

Although LD has the potential to facilitate studies on the excited-state dynamics of large neutral biological molecules, it does have limitations. For example, it is not possible to ensure that there will be no fragmentation of the desorbed molecules. The technique of LIAD directly addresses this problem. The principles of LIAD have been known for decades [86]. However, as a tool for spectroscopists, LIAD is still in its infancy and, as such, there are very few examples of its successful implementation in excited-state dynamics of biomolecules. The principle of LIAD is to use the energy of the desorbing laser to heat a foil (usually titanium or tantalum, see below) on which the analyte has been deposited. The analyte sample is deposited on one side of the foil, while the opposite side is exposed to pulses of light from a laser. The rapid heating of a small area of the foil by the laser creates acoustic waves that propagate through the foil with enough energy to cause desorption of molecules on the opposite side. The acoustic pulse is relatively long lived, when viewed in terms of ultrafast fs experiments, taking ns to propagate through the foil. A typical set-up is illustrated in figure 6*a*; ns UV pulses create the acoustic waves, while the desorbed molecules interact downstream with fs pump and probe pulses for dynamics measurements [89].

Much work has been carried out in recent years on the characterization of the LIAD process [88–90]. There are many factors to consider in the construction of an LIAD experiment, the metal foil substrate used being of critical importance. For the efficient propagation of the acoustic wave through the foil, the pulse duration of the desorbing laser needs to be shorter than the thermal relaxation time (related to the thermal conductivity) of the foil. The repetition rate of the desorbing laser can, however, be of essentially any value so long as this condition is met. Other factors must also be considered including: the melting point of the foil, foil thickness and foil reflectivity. A thicker foil will withstand a higher intensity laser pulse but will cause greater attenuation of the acoustic wave. Generally, optimal foil thicknesses have been found to be tens of micrometres [87,90].

Shea *et al.* [88] performed a detailed analytical study to determine the best metal to use as an LIAD foil substrate. Six different metals were tested (gold, aluminium, copper, titanium, silver and iron) onto which were deposited the tetrapeptide Val-Ala-Ala-Phe (VAAF) via an electrospray process. The second harmonic of an Nd : YAG laser (532 nm), serving as the desorption laser, was focused onto the metal foil substrate to an area of approximately 10^−3^ cm^−2^, while charge was applied to the polypeptide via proton transfer from (pyridine + H)^+^ to make (VAAF + H)^+^. Figure 6*b* plots the signal intensity of the protonated VAAF as a function of the cumulative number of desorption laser shots for each of the six metals. It is clear that titanium produces the strongest (VAAF + H)^+^ signal from the least amount of cumulative laser shots. From the remaining metals tested, only silver and aluminium show significant desorption; however, a large number of cumulative laser shots (1200–1500) are required before any appreciable (VAAF + H)^+^ signal is reached. Recently, it has been demonstrated that tantalum can be used with some success [87,89]. Tantalum has a higher melting point than titanium, although it has the disadvantage that it does not allow as efficient a propagation of the acoustic wave. As such, the most common foils used are titanium and tantalum, with a thickness of 10–25 μm.

Recently, Belshaw *et al.* [87] have successfully coupled LIAD with ultrafast pump–probe spectroscopy, by monitoring the excited-state dynamics of charge migration in the amino acid, phenylalanine. Transfer of electronic charge is an essential process to understand in biological molecules, initiating a wide variety of chemical reactions including DNA damage from UV radiation [91]. In these experiments, the phenylalanine powder was applied to a tantalum foil substrate, 10 μm thick, in microgram quantities. Previous work by the same group had shown that a sample dissolved in methanol and then applied to the foil and allowed to dry, gave large shot-to-shot variations in signal from the desorption laser and only approximately 500 shots before the signal began to significantly deteriorate [89]. By applying the sample in solid crystalline/powder form, rather than the usual drip-dry solution deposition methods, significant improvement on shot-to-shot signal variation and signal longevity was attained. The 20 Hz desorption laser outputted 0.2 mJ in 5 ns at 355 nm. The intensity of the laser at the foil surface was approximately 3×10^8^ W cm^−2^. Once desorbed, the phenylalanine molecules were photoexcited with a 1.5 fs extreme ultraviolet (XUV) pulse (16–40 eV) and subsequently probed at varying time delays using a 6 fs laser pulse, spanning a bandwidth of 500–950 nm (VIS/NIR), with an intensity of 8×10^12^ W cm^−2^. Resulting ions and fragments were then accelerated into a linear TOF-MS. The set-up is schematically shown in figure 6*a*.

**Figure 7.**
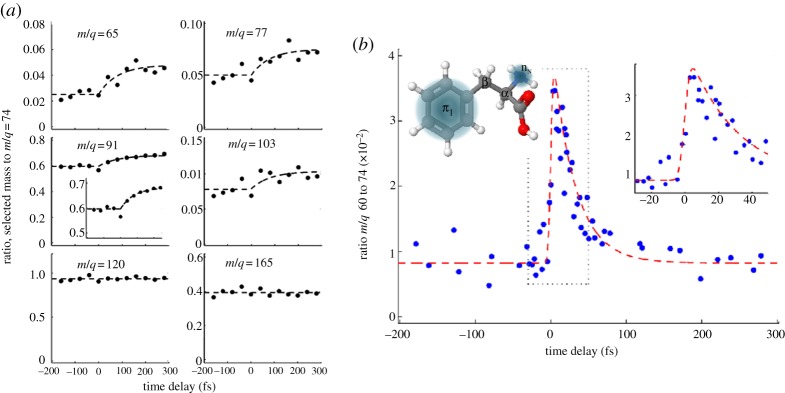
(*a*) Transient parent and fragment cation signals from LIAD study of phenylalanine and (*b*) transient of immonium^2+^ following ionization of the LIAD phenylalanine source. Reproduced with permission from [87].

**Figure 8.**
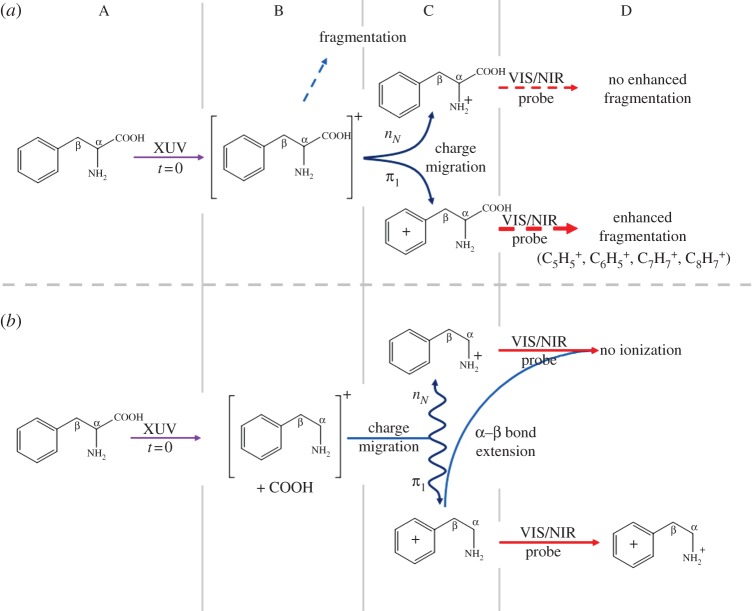
(*a*) Process for time-dependent ion signal in charged ring fragments. A, initial XUV ionization; B, initial fragmentation of charged parent (in this case, the unfragmented parent is followed further); C, charge migration of hole onto either amine or phenyl group; D, probe VIS/NIR pulse applied at some time Δ*t*≥0. If the charge resides on the amine, there is no further fragmentation. If it resides on the phenyl, fragmentation is enhanced in the indicated channels. (*b*) Process for time-dependent ion signal of doubly charged amine. A, initial XUV ionization; B, initial fragmentation to immonium cation; C, charge migration. Hole oscillates between amine and phenyl site. As the C_*α*_–C_*β*_ bond stretches the charge preferentially resides on the amine. D, probe VIS/NIR pulse applied at some time Δ*t*≥0. If the charge resides on the amine, there is no further ionization, if it resides on the phenyl, the ionization occurs leaving a hole on both the amine and phenyl sites.

The structure of phenylalanine is shown in the inset in figure 7*b*. The similarity in electron binding energy between the amine and phenyl functional groups (highlighted in the inset) in the radical cation makes it an ideal molecule for the study of charge migration between these charge acceptor sites. Figure 7*a* shows transients of the parent cation (*m*/*q*=165) and a series of selected fragments. The parent cation and immonium fragment (*m*/*q*=120) transients show no time dependence. The remaining fragments however, at *m*/*q*=65, 77, 91 and 103, all display a rise in relative abundance after time zero (Δ*t*=0) with appearance times that are consistent, leading to a weighted average time constant of 80±20 fs. These fragments all correspond to charge residing on the phenyl group demonstrating a motion of the charge towards this group over time, following ionization with the XUV pulse. The mechanism suggested for this charge migration is IC of this charge onto the *π*_1_ state of the phenyl group in the radical cation. This state is known to absorb strongly in the visible (this is not the case for the neutral phenyl or amine, nor for the charged amine [92]). The resonant one-photon transitions induced by the VIS/NIR probe thus enhance the production of fragments where the charge is localized on the phenyl group. This process is further elucidated in figure 8*a*.

Whereas the singly charged immonium cation (loss of COOH group) shows no time-dependent behaviour, the immonium dication (*m*/*q*=60) does. The loss of the COOH group stabilizes the resulting immonium cation, allowing a second ionization event after which electron holes reside on both the amine and phenyl groups. This ion fragment is particularly sensitive to charge migration, as the charge concentrated on the amine group leads to a rise in the local ionization potential, suppressing further ionization from the *n*_*N*_ orbital localized on the amine by the VIS/NIR probe laser. Figure 7*b* shows the time-dependent behaviour of the immonium dication. There is a sharp rise around time zero followed by a rapid decay with a time constant of approximately 30 fs. Belshaw *et al.* proposed that this decay corresponds to the time scale for nuclear rearrangement. Small-scale oscillations in the signal close to time zero are attributed to the charge oscillating between the ring and amine sites before a loss of coherence of the electron wavepacket is brought about by bond stretching, leaving the charge finally residing on the amine site. The pathways for this process are illustrated in figure 8*b*.

These aforementioned studies demonstrate that LIAD can be successfully used to generate a sufficient number density of large neutral biological molecules in the gas phase (when directly probing the plume, although still relatively low compared with LD) with minimal fragmentation and no direct ionization from the desorption laser. While the amalgamation of LIAD with ultrafast gas-phase spectroscopy is still in its infancy, there are nonetheless a growing number of groups that are more generally using the technique to perform gas-phase experiments [93–95]. Whereas LIAD has the advantage of producing isolated gas-phase neutrals, the main drawback is the low sample density in the desorbed plume. This makes coupling LIAD with other techniques such as free-jet expansions for molecular cooling very difficult as only a small proportion (less than 1%) of the desorbed molecules will be picked up by the expanded gas pulse. Careful application of the analyte on the substrate can help to improve the sample density; however, there is still a great deal of developmental work to be done on LIAD, making it very promising for future studies of a range of biomolecules.

### (c) Excited-state dynamics using electrospray ionization

**Figure 9.**
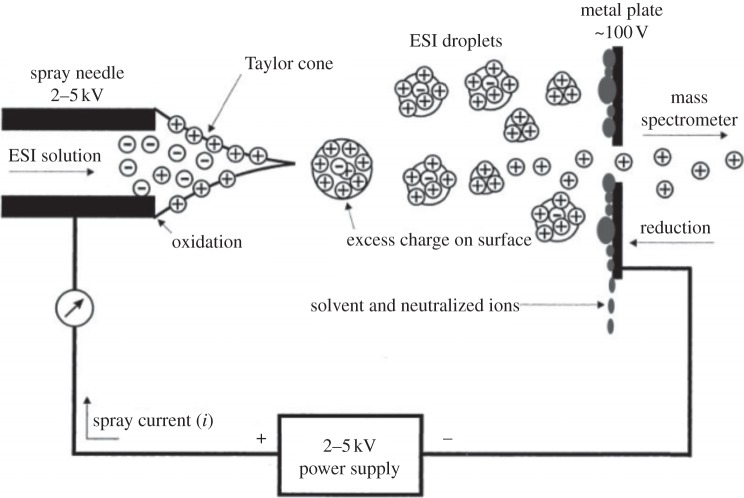
Diagram of the ESI process. Reproduced with permission from [102].

There has been prominent success in probing the excited-state dynamics of DNA bases and aromatic amino acid chromophores. In addition, we have seen the promising potential of LD and LIAD in furthering these measurements to larger systems. More success however, has been realized in the study of excited-state dynamics of charged biomolecules. This is an important field of study, as molecules in cells often exist in a charged state or environment [96]. Electrospray ionization (ESI) is a technique that was first pioneered in the late 1960s by Dole [97,98] and later advanced by Fenn [99]. It is an extremely versatile method of introducing molecules of seemingly unlimited size into the gas phase. Samples as large as viruses have successfully been vaporized and studied using this technique [100,101]. In this method, a solution of molecules is flowed through a syringe, the needle of which terminates at a capillary. The capillary and the instrument inlet are kept at some potential difference, usually kilovolts (depending on the solvent used). The sample is pushed through the syringe, typically at microlitre flow rates, into a region held at atmosphere, forming highly charged droplets of the sample molecule in solution. As the sample expands into this region, the solvent evaporates, increasing charge density within the droplets until a coulombic explosion occurs. A buffer gas may also be passed over the expanding spray to remove the solvent vapour from the sample droplets, as well as for the purposes of collisional cooling. The sample ions are then expanded into an evacuated region (approx. 10^−3^mbar), which houses the ion optics/detection systems, and then pass through a further series of differentially pumped vacuum regions to attain a pressure appropriate for the experiment being performed (figure 9). As in ESI, the molecules of interest begin in a solvated state, it is a relatively simple affair to produce, isolate and study microsolvated isolated molecules using this technique [103]. Such studies offer valuable insights into the phase-specific dynamics of biomolecules, bridging the gap between the gas and bulk solution phases [104].

As noted above, ESI has been successfully coupled with ultrafast pump–probe spectroscopy to study the excited-state dynamics of biomolecules. Notable examples include protonated aromatic amino acids and dipeptides [105–107], protonated nucleobases [108] and deprotonated nucleotides [109]. For the purpose of the ensuing discussion, we begin with a description of the work by Kang *et al.* [105] on the protonated aromatic amino acids phenylalanine (Phe), tryptophan (Trp) and tyrosine (Tyr), the structures of which are shown in figure 1*c*.

In their experiment, Kang *et al.* [105] coupled an ESI source, in which an amino acid in a 1 : 1 water–methanol solution (500 μmol) was sprayed through a stainless steel needle valve towards the entrance of a desolvation capillary biased at +4 kV. The ions then entered the TOF-MS through a series of ion optics including a hexapole ion guide, a pulsed exit lens and a set of electrostatic lenses. The exit lens held the cations in the hexapole for 1 ms before being ejected for 800 ns, focused and extracted towards the field-free TOF-MS. The pump pulse (266 nm, 50–100 μJ pulse^−1^) was the third harmonic of a Ti : sapphire laser operating at 1 kHz, while the probe pulse was the fundamental 800 nm radiation (150 μJ pulse^−1^). In order to probe the excited-state dynamics of the protonated amino acids, the pump pulse photoexcited the amino acid to the ^1^ππ* state. By probing the variation in the side chain fragment intensity (via C_*α*_–C_*β*_ cleavage, cf. figure 7*b*) as a function of pump–probe delay, this directly reflects the variation in fragment intensity obtained through initially exciting the ^1^ππ* state of the amino acids with the pump pulse and the final state accessed with the probe pulse.

Following the method described earlier, a lifetime was extracted for the TrpH^+^^1^ππ* state of 380±50 fs and for TyrH^+^ of 22.3±1 ps. From time-dependent density functional theory calculations, Kang *et al.* suggested that these lifetimes were because of a charge transfer from the initially excited ^1^ππ* state to a dissociative state along the N–H coordinate of the amine group with σ* character. The near order of magnitude difference between the lifetimes was attributed to a difference in barrier height produced by the ^1^ππ*/^1^πσ* CI (figure 2). It should be noted, however, that improvements in experimental design allowed the group to isolate a biexponential decay in the *m*/*q* = 130 fragment of TrpH^+^, producing a second lifetime of approximately 15 ps [110]. This was attributed to either conformer-specific dynamics or a population transfer from the σ* state to a π* state located on the carboxylic acid group (labelled 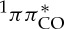), the 15 ps value then corresponding to the lifetime of this state.

**Figure 10.**
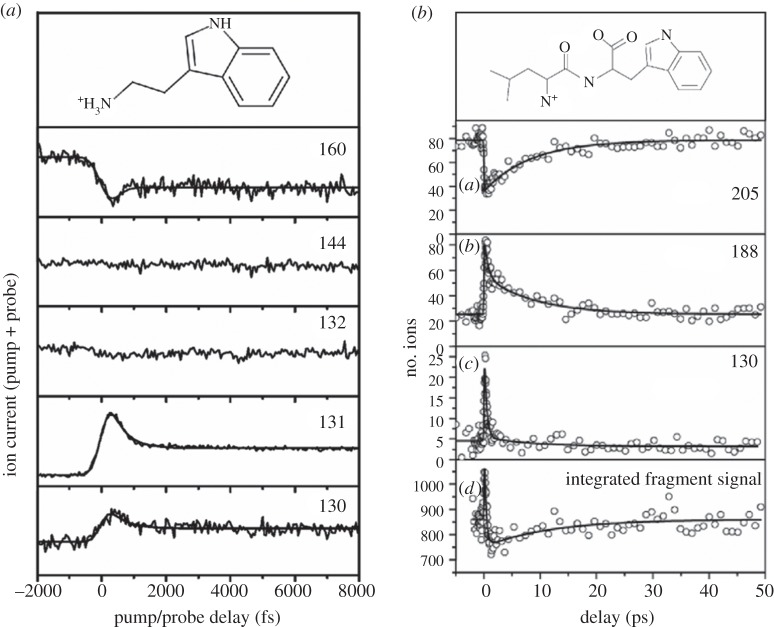
(*a*) Time-dependent mass channel signals in an ESI study of TrypH^+^. Reproduced with permission from [106]. (*b*) Time-dependent mass channel signals in an electrospray ionization study of LWH^+^. Reproduced with permission from [111].

Kang *et al.* [106] extended these measurements to study the protonated derivative of tryptophan, tryptamine (TrypH^+^) shown in the top panel of figure 10*a*. Unlike their earlier work, where only one of the fragment channels was probed, these studies looked at a range of fragment channels. Figure 10*a* shows a range of transients recorded by probing the deprotonated cation (Tryp^+^) at *m*/*q*=160 and fragments at 144, 132, 131 and 130. For the *m*/*q*=131 and 130 fragments, pump–probe dynamics are clearly observed, the signal showing a fast rise at Δ*t*=0 followed by a decay to a plateau with a time constant of approximately 250 fs. An equivalent depletion followed by a rise to a plateau is demonstrated in the Tryp^+^ transient, suggesting H-atom loss is driving all three processes. The remaining fragments (*m*/*q*=144 and 132) showed no apparent dynamics, indicating an alternative, one-colour fragmentation process.

Kang *et al.* proposed two competing pathways to explain these results; IC to a highly vibrationally excited ground state or H-atom loss from the amino functional group. The H-atom loss is rationalized by the now familiar coupling of the initially excited ^1^ππ* state with a dissociative ^1^πσ* state (cf. figure 2 schematic) along the NH coordinate of the amino group. This coupling allows for rapid H-loss along the repulsive ^1^πσ* potential surface, which accounts for the very short lifetime of the initially excited ^1^ππ* state of approximately 250 fs. Importantly, once on the ^1^πσ* state, the system evolves towards the 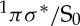 CI, leading to a branching in the excited-state flux, resulting in either H-atom loss to generate Tryp^+^ or repopulation of TrypH^+^ in S_0_. Owing to low-lying electronic states in Tryp^+^, these can be excited with the 800 nm probe leading to subsequent dissociation to yield fragments at *m*/*q*=131 and 130, ergo the similar time constants observed for *m*/*q*=160, 131 and 130. Repopulation of TrypH^+^ in S_0_ has a closed shell electronic structure and does not absorb the 800 nm probe. This IC mechanism, however, leaves TrypH^+^ with large amounts of excess internal (vibrational) energy in S_0_, leading to the two fragments at *m*/*q*=144 and 132, which do not display time dependence.

Nolting *et al.* [111] extended these measurements to the protonated dipeptide H_2_N-Leu-Trp-COOH^+^ (LWH^+^) shown in the top panel of figure 10*b*. The experimental set-up for this work was very similar to that outlined above. Once again, a Ti : sapphire 1 kHz laser was used; the pump pulse was the third harmonic of the fundamental (266 nm, 750 nJ pulse^−1^) while the probe was once again the fundamental 800 nm radiation (100 μJ pulse^−1^). For each measurement, approximately 500 pump–probe pulse pairs interacted with the same ensemble of ions. This corresponded to approximately 500 mass selected ions, which were allowed to accumulate within an ion trap, leading to a fragmentation rate of approximately 2. At the repetition rate of the experiment, this proved to be more than sufficient to produce discernible signals.

Mass fragmentation channels of *m*/*q*=205, 188 and 130 were focused on for this study, and the transients are presented in figure 10*b*. The transients indicate that there are two clear time regimes being probed. The *m*/*q*=205 mass fragment, which corresponds to the loss of the amino acid leucine (Leu), shows an initial depletion step followed by a recovery with a time constant of approximately 9 ps. The *m*/*q*=188 (loss of Leu and NH_3_) fragment decays with two time constants. The first corresponds to approximately 550 fs, whereas the second is approximately 8 ps, which matches the recovery of the fragment at *m*/*q*=205. Finally, the peak at *m*/*q*=130 (loss of multiple neutral subunits) shows only the ultrafast (subpicosecond) behaviour, with an associated exponential decay of approximately 430 fs.

Similar to the conclusions drawn by Kang *et al.* [110] regarding TrpH^+^, Nolting *et al*. [111] suggested two possible explanations for the biexponential behaviour of LWH^+^; conformer-specific dynamics or sequential IC. Focusing on the second of these possibilities, it is the fragmentation behaviour, as was the case in TrypH^+^ above, that yielded the clues to understanding this mechanism. Nolting *et al.* suggested that, as both the S_1_ and S_2_ states (both of ^1^ππ* character) of LWH^+^ are readily accessible by the pump photon, IC is occurring between the two on an ultrafast time scale (hundreds of fs) with IC from the S_1_ state back to the ground state then occurring over ps (perhaps akin to the 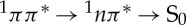 relaxation pathway observed in isolated DNA bases, see introduction, or the 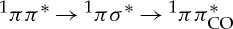 dynamics observed in TrpH^+^ [110]). Different higher excited states would likely be accessible from the S_1_ and S_2_ states owing to their differing absorption cross sections at the probe wavelength of 800 nm (i.e. the S_2_ state may be assumed to have a small 800 nm absorption cross section, only allowing single photon absorption to higher states, whereas a larger absorption cross section in S_1_ would allow multi-photon absorption to even higher lying states), resulting in different fragmentation intensities upon probing, and hence the dynamics observed. While conformer-specific dynamics are also cited as a possible solution, and although it is known in general that different conformers have the potential to exhibit different dynamics [112–115], no explicit photochemical explanation is offered as to why these time constants would be orders of magnitude different. It is of significance to note here, that routinely monitoring isolated conformer-specific dynamics in the time domain to this day still remains somewhat of a ‘holy grail’ within the molecular dynamics community.

While a great deal of spectroscopy and spectrometry has been performed on peptides using ESI, the application of this technique to study the dynamics of DNA and its substituents is still very much in its infancy. Chatterley *et al.* [109] have performed experiments on the deprotonated nucleotide anions of all four DNA bases by coupling ESI with TR-PES using an experimental set-up described in detail separately [116]. In this work, dXMP^−^ (where X is a DNA base, for example, dGMP^−^ would be 2′deoxy-guanosine 5′-monophosphate, see figure 1*b*) was generated via ESI from an approximately 0.5 mM solution of either dXMP or its sodium salt in methanol. Deprotonated dXMP^−^ anions were then stored in a trap for approximately 200 ms and pulsed collinearly into a TOF-MS. 267 nm pulses (5–10 μJ pulse^−1^), loosely focused and resonant with the ^1^ππ* state, then intersected the ion packet and the emitted photoelectrons were analysed according to the electron kinetic energy using VMI (see §2*c*).

**Figure 11.**
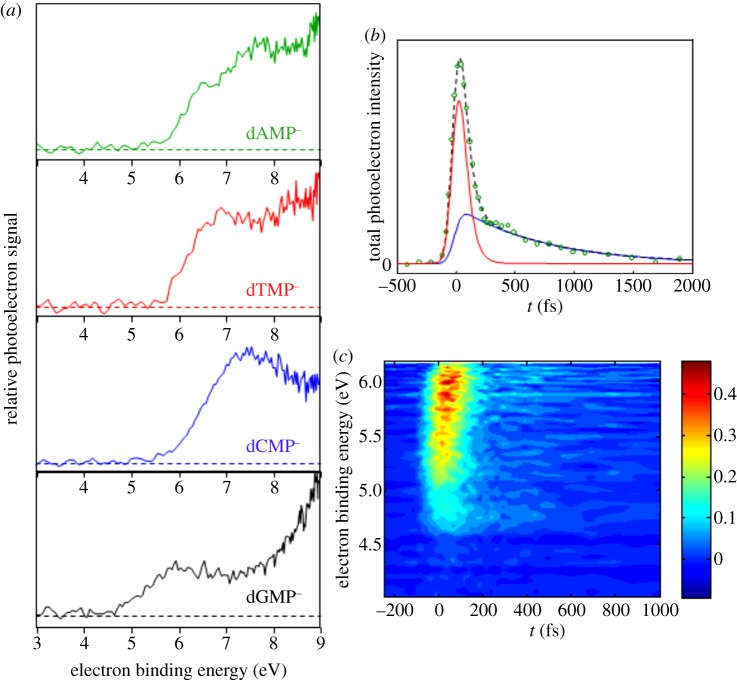
(*a*) Two-photon resonant photodetachment spectra of (top–bottom) dAMP^−^, dTMP^−^, dCMP^−^ and dGMP^−^, (*b*) time-resolved total electron yield of dGMP^−^ fit to two exponential decays yielding lifetimes of less than 50 fs (red) and approximately 700 fs (blue) and (*c*) time-resolved photoelectron spectrum of dGMP^−^. Reproduced with permission from [109].

Figure 11*a* shows resonant two-photon photodetachment spectra for all four nucleotide anions. While dAMP^−^, dTMP^−^ and dCMP^−^ all show detachment onsets of approximately 5.7 eV, dGMP^−^ shows an onset that is considerably lower in energy, corresponding to approximately 4.7 eV. Previous one-photon detachment work at 157 nm by Yang *et al.* [117] demonstrated a lower electron binding onset for dAMP^−^, dTMP^−^ and dCMP^−^, whereas their results for dGMP^−^ were comparable to the results presented in figure 11*a*. In their experiment, Yang *et al.* [117] were able to one-photon ionize from the sugar and the phosphate as well as the base, whereas Chatterley *et al.* could only resonantly two-photon ionize the base. As such, in the work by Yang *et al.*, the lowest electron binding energy in dAMP^−^, dTMP^−^ and dCMP^−^ was attributed to photodetachment from the phosphate moiety, whereas for dGMP^−^, the lowest binding energy site corresponds to photodetachment from the base moiety, in agreement with Chatterley *et al.* [109].

Chatterley *et al.* extended these measurements and performed preliminary pump–probe measurements on dGMP^−^. In these experiments, the pump pulse was once again centred at 267 nm, while the probe pulse was the fundamental 800 nm radiation (200–300 μJ pulse^−1^). Figure 11*b*,*c* shows the time-resolved total photoelectron yield and time/energy-resolved photoelectron spectrum, respectively. The transient from the total photoelectron signal was modelled with two decay functions, yielding time constants of approximately 50 and 700 fs. The approximately 50 fs decay and associated blue shift in the electron binding energy were attributed to photodetachment from both the initially populated ^1^ππ* state and lower lying states. These dynamics are the cause of the spectral shapes observed in figure 11*a*, as these are one-colour spectra that will involve a convolution of data from all points in time within the pulse width of the laser (approx. 120 fs [109]). Further experiments are currently underway to rationalize the origin of these time constants more fully. The longer time constant (700 fs) was not further explored in this preliminary work by Chatterley *et al.*, as the exact derivations of these dynamics remain under debate.

The keen observer might note at this point that no examples have been given involving the use of TR-VMI (see §2) to study the time evolution of photoelectron angular distributions. It should be noted that such a time-resolved study that couples an electrospray source to a VMI spectrometer does exist [118]. However, no examples exist to date involving biomolecules as the analyte, although there is no reason why this cannot be done now that successful coupling of ESI with TR-VMI has been demonstrated.

## 4. Summary and outlook

The work outlined in this review demonstrates the seemingly boundless potential that is only now being realized in performing pump–probe experiments on large, biologically relevant molecules in the gas phase and the significance that these experiments hold to our understanding as to why natural selection led to specific sets of molecular building blocks. With the recent innovations in gas-phase technology outlined above, this allows a much more diverse range of isolated biomolecules to be studied in vacuum, including the tantalizing prospect of performing microsolvation studies [119,120]. As such, this opens the door for a greater understanding of dynamics in more complex environments, akin to the environment in which these molecules naturally exist.

One important step for future consideration will be to investigate how the dynamics in vacuum translate into the solution phase; the phase in which biological systems are most usually found. Early experiments in this direction have shown that the knowledge gleaned from measurements in vacuum may have a very promising bearing on the dynamics in the solution phase, particularly in weakly interacting solvents. While these comparative studies are still in their infancy [24,121], the future of excited-state dynamics of biomolecules in the gas phase is bright.

## References

[1] Eberhard S Finazzi G Wollman FA 2008 The dynamics of photosynthesis. Annu. Rev. Genet. 42, 463–515 (doi:10.1146/annurev.genet.42.110807.091452) 18983262 10.1146/annurev.genet.42.110807.091452

[2] Collini E Wong CY Wilk KE Curmi PM Brumer P Scholes GD 2010 Coherently wired light-harvesting in photosynthetic marine algae at ambient temperature. Nature 463, 644–677 (doi:10.1038/nature08811) 20130647 10.1038/nature08811

[3] Lee H Cheng YC Fleming GR 2007 Coherence dynamics in photosynthesis: protein protection of excitonic coherence. Science 316, 1462–1465 (doi:10.1126/science.1142188) 17556580 10.1126/science.1142188

[4] Cheng YC Fleming GR 2009 Dynamics of light harvesting in photosynthesis. Annu. Rev. Phys. Chem. 60, 241–262 (doi:10.1146/annurev.physchem.040808.090259) 18999996 10.1146/annurev.physchem.040808.090259

[5] Arnett DC Moser CC Dutton PL Scherer NF 1999 The first events in photosynthesis: electronic coupling and energy transfer dynamics in the photosynthetic reaction center from Rhodobacter sphaeroides. J. Phys. Chem. B 103, 2014–2032 (doi:10.1021/jp984464j)

[6] Harris DL 2002 Oxidation and electronic state dependence of proton transfer in the enzymatic cycle of cytochrome P450eryF. J. Inorg. Biochem. 91, 568–585 (doi:10.1016/s0162-0134(02)00477-4) 12237223 10.1016/s0162-0134(02)00477-4

[7] Horenstein BA Schramm VL 1993 Electronic nature of the transition state for nucleoside hydrolase. A blueprint for inhibitor design. Biochemistry 32, 7089–7097 (doi:10.1021/bi00079a004) 8343502 10.1021/bi00079a004

[8] Middleton CT de La Harpe K Su C Law YK Crespo-Hernandez CE Kohler B 2009 DNA excited-state dynamics: from single bases to the double helix. Annu. Rev. Phys. Chem. 60, 217–239 (doi:10.1146/annurev.physchem.59.032607.093719) 19012538 10.1146/annurev.physchem.59.032607.093719

[9] Crespo-Hernandez CE Cohen B Hare PM Kohler B 2004 Ultrafast excited-state dynamics in nucleic acids. Chem. Rev. 104, 1977–2019 (doi:10.1021/cr0206770) 15080719 10.1021/cr0206770

[10] Sinha RP Häder D-P 2002 UV-induced DNA damage and repair: a review. Photochem. Photobiol. Sci. 1, 225–236 (doi:10.1039/b201230h) 12661961 10.1039/b201230h

[11] Rossle S Friedrichs J Frank I 2010 The formation of DNA photodamage: the role of exciton localization. Chem. Phys. Chem. 11, 2011–2015 (doi:10.1002/cphc.201000081) 20449863 10.1002/cphc.201000081

[12] Bisgaard CZ Satzger H Ullrich S Stolow A 2009 Excited-state dynamics of isolated DNA bases: a case study of adenine. Chem. Phys. Chem. 10, 101–110 (doi:10.1002/cphc.200800516) 19009581 10.1002/cphc.200800516

[13] Roberts GM Stavros VG 2013 *πσ** mediated dynamics in heteroatomic biomolecules and their subunits: insights from gas phase femtosecond spectroscopy. In preparation

[14] Ullrich S Schultz T Zgierski MZ Stolow A 2004 Electronic relaxation dynamics in DNA and RNA bases studied by time-resolved photoelectron spectroscopy. Phys. Chem. Chem. Phys. 6, 2796–2801 (doi:10.1039/b316324e) 10.1021/ja030532q 14982403

[15] Ashfold MN King GA Murdock D Nix MG Oliver TA Sage AG 2010 ps* excited states in molecular photochemistry. Phys. Chem. Chem. Phys. 12, 1218–1238 (doi:10.1039/b921706a) 20119599 10.1039/b921706a

[16] Satzger H Townsend D Zgierski MZ Patchkovskii S Ullrich S Stolow A 2006 Primary processes underlying the photostability of isolated DNA bases: adenine. Proc. Natl Acad. Sci. USA 103, 10 (doi:10.1073/pnas.0602663103) 16803961 10.1073/pnas.0602663103 PMC1502434

[17] Wells KL Hadden DJ Nix MGD Stavros VG 2010 Competing *π σ** states in the photodissociation of adenine. J. Phys. Chem. Lett. 1, 993–996 (doi:10.1021/jz100050y)

[18] Canuel C Mons M Piuzzi F Tardivel B Dimicoli I Elhanine M 2005 Excited states dynamics of DNA and RNA bases: characterization of a stepwise deactivation pathway in the gas phase. J. Chem. Phys. 122, 074316 (doi:10.1063/1.1850469) 15743241 10.1063/1.1850469

[19] Roberts GM Chatterley AS Young JD Stavros VG 2012 Direct observation of hydrogen tunneling dynamics in photoexcited phenol. J. Phys. Chem. Lett. 3, 348–352 (doi:10.1021/jz2016318) 10.1021/jz2016318 26285849

[20] Perun S Sobolewski AL Domcke W 2005 Ab initio studies on the radiationless decay mechanisms of the lowest excited singlet states of 9H-adenine. J. Am. Chem. Soc. 127, 6257–6265 (doi:10.1021/ja044321c) 15853331 10.1021/ja044321c

[21] Kleinermanns K Nachtigallová D de Vries MS 2013 Excited state dynamics of DNA bases. Int. Rev. Phys. Chem. 32, 308–342 (doi:10.1080/0144235x.2012.760884)

[22] Pino GA 2010 Excited state hydrogen transfer dynamics in substituted phenols and their complexes with ammonia: pp*-ps* energy gap propensity and ortho-substitution effect. J. Chem. Phys. 133, 124313 (doi:10.1063/1.3480396) 20886938 10.1063/1.3480396

[23] Dixon RN Oliver TA Ashfold MN 2011 Tunnelling under a conical intersection: application to the product vibrational state distributions in the UV photodissociation of phenols. J. Chem. Phys. 134, 194303 (doi:10.1063/1.3585609) 21599055 10.1063/1.3585609

[24] Zhang Y Oliver TAA Ashfold MNR Bradforth SE 2012 Contrasting the excited state reaction pathways of phenol and para-methylthiophenol in the gas and liquid phases. Faraday Discuss. 157, 141–163 (doi:10.1039/c2fd20043k) 23230767 10.1039/c2fd20043k

[25] Sobolewski AL Domcke W Dedonder-Lardeux C Jouvet C 2002 Excited-state hydrogen detachment and hydrogen transfer driven by repulsive ^1^*π σ** states: a new paradigm for non-radiative decay in aromatic biomolecules. Phys. Chem. Chem. Phys. 4, 1093–1100 (doi:10.1039/b110941n)

[26] Nir E Plützer C Kleinermanns K de Vries M 2002 Properties of isolated DNA bases, base pairs and nucleosides examined by laser spectroscopy. Eur. Phys. J. D. 20, 317–329 (doi:10.1140/epjd/e2002-00167-2)

[27] Campargue R 1984 Progress in overexpanded supersonic jets and skimmed molecular beams in free-jet zones of silence. J. Phys. Chem. 88, 4466–4474 (doi:10.1021/j150664a004)

[28] Scoles G 1988 Atomic and molecular beam methods. Oxford, UK: Oxford University Press

[29] Wiley WC McLaren IH 1955 Time-of-flight mass spectrometer with improved resolution. Rev. Sci. Instrum. 26, 1150 (doi:10.1063/1.1715212)

[30] Kang H Lee KT Jung B Ko YJ Kim SK 2002 Intrinsic lifetimes of the excited state of DNA and RNA bases. J. Am. Chem. Soc. 124, 12 (doi:10.1021/ja027627x) 12405817 10.1021/ja027627x

[31] Samoylova E Lippert H Ullrich S Hertel IV Radloff W Schultz T 2005 Dynamics of photoinduced processes in adenine and thymine base pairs. J. Am. Chem. Soc. 127, 1782–1786 (doi:10.1021/ja044369q) 15701013 10.1021/ja044369q

[32] Iqbal A Pegg LJ Stavros VG 2008 Direct versus indirect H atom elimination from photoexcited phenol molecules. J. Phys. Chem. A 112, 9531–9534 (doi:10.1021/jp802155b) 18540588 10.1021/jp802155b

[33] Montero R Conde AP Ovejas V Castano F Longarte A 2012 Ultrafast photophysics of the isolated indole molecule. J. Phys. Chem. A 116, 2698–2703 (doi:10.1021/jp207750y) 22050115 10.1021/jp207750y

[34] Montero R Peralta Conde A Ovejas V Fernandez-Fernandez M Castano F Vazquez de Aldana JR Longarte A 2012 Femtosecond evolution of the pyrrole molecule excited in the near part of its UV spectrum. J. Chem. Phys. 137, 064317 (doi:10.1063/1.4742344) 22897283 10.1063/1.4742344

[35] Lippert H Ritze HH Hertela IV Radloff W 2004 Femtosecond time-resolved hydrogen-atom elimination from photoexcited pyrrole molecules. Chem. Phys. Chem. 5, 1423–1427 (doi:10.1002/cphc.200400079) 15499860 10.1002/cphc.200400079

[36] Roberts GM Williams CA Yu H Chatterley AS Young JD Ullrich S Stavros VG 2013 Probing ultrafast dynamics in photoexcited pyrrole: timescales for ^1^*π σ** mediated H-atom elimination. Faraday Discuss 163, 95–116 (doi:10.1039/c2fd20140b) 24020198 10.1039/c2fd20140b

[37] Montero R Peralta A Conde Ovejas V Fernandez-Fernandez M Castano F Longarte A 2012 Ultrafast evolution of imidazole after electronic excitation. J. Phys. Chem. A 116, 10 (doi:10.1021/jp3078198) 10.1021/jp3078198 23088353

[38] Yu H Evans NL Stavros VG Ullrich S 2012 Investigation of multiple electronic excited state relaxation pathways following 200 nm photolysis of gas-phase imidazole. Phys. Chem. Chem. Phys. 14, 6266–6272 (doi:10.1039/c2cp23533a) 22370631 10.1039/c2cp23533a

[39] Vallance C 2004 ‘Molecular photography’: velocity-map imaging of chemical events. Phil. Trans. R. Soc. Lond. A 362, 2591–2609 (doi:10.1098/rsta.2004.1460) 10.1098/rsta.2004.1460 15539360

[40] Greaves SJ Rose RA Orr-Ewing AJ 2010 Velocity map imaging of the dynamics of bimolecular chemical reactions. Phys. Chem. Chem. Phys. 12, 9129–9143 (doi:10.1039/c001233e) 20448868 10.1039/c001233e

[41] Reid KL 2003 Photoelectron angular distributions. Annu. Rev. Phys. Chem. 54, 397–424 (doi:10.1146/annurev.physchem.54.011002.103814) 12574491 10.1146/annurev.physchem.54.011002.103814

[42] Stolow A Bragg AE Neumark DM 2004 Femtosecond time-resolved photoelectron spectroscopy. Chem. Rev. 104, 1719–1757 (doi:10.1021/cr020683w) 15080710 10.1021/cr020683w

[43] Eppink ATJB Parker DH 1997 Velocity map imaging of ions and electrons using electrostatic lenses: application in photoelectron and photofragment ion imaging of molecular oxygen. Rev. Sci. Instrum. 68, 3477 (doi:10.1063/1.1148310)

[44] Cavanagh S Gibson S Gale M Dedman C Roberts E Lewis B 2007 High-resolution velocity-map-imaging photoelectron spectroscopy of the O^−^ photodetachment fine-structure transitions. Phys. Rev. A 76, 052708 (doi:10.1103/PhysRevA.76.052708)

[45] Iqbal A Cheung MS Nix MG Stavros VG 2009 Exploring the time-scales of H-atom detachment from photoexcited phenol-h_6_ and phenol-d_5_: statistical vs nonstatistical decay. J. Phys. Chem. A 113, 8157–8163 (doi:10.1021/jp9031223) 19569697 10.1021/jp9031223

[46] Iqbal A Stavros VG 2010 Exploring the time scales of H-atom elimination from photoexcited indole. J. Phys. Chem. A 114, 68–72 (doi:10.1021/jp908195k) 19928773 10.1021/jp908195k

[47] Hadden DJ Wells KL Roberts GM Bergendahl LT Paterson MJ Stavros VG 2011 Time resolved velocity map imaging of H-atom elimination from photoexcited imidazole and its methyl substituted derivatives. Phys. Chem. Chem. Phys. 13, 10 (doi:10.1039/c1cp20463g) 10.1039/c1cp20463g 21512683

[48] Livingstone RA Thompson JO Iljina M Donaldson RJ Sussman BJ Paterson MJ Townsend D 2012 Time-resolved photoelectron imaging of excited state relaxation dynamics in phenol, catechol, resorcinol, and hydroquinone. J. Chem. Phys. 137, 184304 (doi:10.1063/1.4765104) 23163368 10.1063/1.4765104

[49] Evans NL Ullrich S 2010 Wavelength dependence of electronic relaxation in isolated adenine using UV femtosecond time-resolved photoelectron spectroscopy. J. Phys. Chem. A 114 (doi:10.1021/jp1029097) 10.1021/jp1029097 20961159

[50] Spesyvtsev R Kirkby OM Vacher M Fielding HH 2012 Shedding new light on the role of the Rydberg state in the photochemistry of aniline. Phys. Chem. Chem. Phys. 14, 9942–9947 (doi:10.1039/c2cp41785e) 22710758 10.1039/c2cp41785e

[51] Spesyvtsev R Kirkby OM Fielding HH 2012 Ultrafast dynamics of aniline following 269–238 nm excitation and the role of the S2(*π*3s/*π σ**) state. Faraday Discuss. 157, 165–179 (doi:10.1039/c2fd20076g) 23230768 10.1039/c2fd20076g

[52] Livingstone R Schalk O Boguslavskiy AE Wu G Bergendahl LT Stolow A Paterson MJ Townsend D 2011 Following the excited state relaxation dynamics of indole and \hbox5-hydroxyindole using time-resolved photoelectron spectroscopy. J. Chem. Phys. 135, 194307 (doi:10.1063/1.3659231) 22112082 10.1063/1.3659231

[53] Wu G Hockett P Stolow A 2011 Time-resolved photoelectron spectroscopy: from wavepackets to observables. Phys. Chem. Chem. Phys. 13, 18 (doi:10.1039/c1cp22031d) 21947027 10.1039/c1cp22031d

[54] Stolow A 2003 Femtosecond time-resolved photoelectron spectroscopy of polyatomic molecules. Annu. Rev. Phys. Chem. 54, 89–119 (doi:10.1146/annurev.physchem.54.011002.103809) 12524428 10.1146/annurev.physchem.54.011002.103809

[55] Suzuki T 2006 Femtosecond time-resolved photoelectron imaging. Annu. Rev. Phys. Chem. 57, 555–592 (doi:10.1146/annurev.physchem.57.032905.104601) 16599821 10.1146/annurev.physchem.57.032905.104601

[56] Neumark DM 2001 Time-resolved photoelectron spectroscopy of molecules and clusters. Annu. Rev. Phys. Chem. 52, 255–277 (doi:10.1146/annurev.physchem.52.1.255) 11326066 10.1146/annurev.physchem.52.1.255

[57] Kruit P Read FH 1983 Magnetic field paralleliser for 2*π* electron-spectrometer and electron-image magnifier. J. Phys. E 16, 313–324 (doi:10.1088/0022-3735/16/4/016)

[58] Bordas C Paulig F Helm H Huestis DL 1996 Photoelectron imaging spectrometry: principle and inversion method. Rev. Sci. Instrum. 67, 2257 (doi:10.1063/1.1147044)

[59] Koopmans T 1934 Über die Zuordnung von Wellenfunktionen und Eigenwerten zu den Einzelnen Elektronen Eines Atoms. Physica 1, 104–113 (doi:10.1016/s0031-8914(34)90011-2)

[60] Ghafur O Siu W Johnsson P Kling MF Drescher M Vrakking MJ 2009 A velocity map imaging detector with an integrated gas injection system. Rev. Sci. Instrum. 80, 033110 (doi:10.1063/1.3085799) 19334910 10.1063/1.3085799

[61] Wang L-S Reutt-Robey JE Niu B Lee YT Shirley DA 1990 High temperature and high resolution UV photoelectron spectroscopy using supersonic molecular beams. J. Electron Spectrosc. Relat. Phenom. 51, 513–526 (doi:10.1016/0368-2048(90)80177-c)

[62] Smits M de Lange CA Ullrich S Schultz T Schmitt M Underwood JG Shaffer JP Rayner DM Stolow A 2003 Stable kilohertz rate molecular beam laser ablation sources. Rev. Sci. Instrum. 74, 4812 (doi:10.1063/1.1614879)

[63] Hankin SM John P 1999 Microscopic cluster formation during the laser desorption of chrysene-d12. J. Phys. Chem. B. 103, 4566–4569 (doi:10.1021/jp990950a)

[64] Wilson MS McCloskey JA 1975 Chemical ionization mass spectrometry of nucleosides. Mechanisms of ion formation and estimations of proton affinity. J. Am. Chem. Soc. 97, 3436–3444 (doi:10.1021/ja00845a026) 1141575 10.1021/ja00845a026

[65] Vastola FJ Pirone AJ 1968 Ionization of organic solids by laser irradiation. Adv. mass spectrom 4, 107–111

[66] Posthumus MA Kistemaker PG Meuzelaar HLC Ten Noever de Brauw MC 1978 Laser desorption-mass spectrometry of polar nonvolatile bio-organic molecules. Anal. Chem. 50, 985–991 (doi:10.1021/ac50029a040)

[67] Abo-riziq A Crews BO Compagnon I Oomens J Meijer G Von Helden G Kabelac M Hobza P de Vries MS 2007 The mid-IR spectra of 9-ethyl guanine, guanosine, and 2-deoxyguanosine. J. Phys. Chem. A 111, 7529–7536 (doi:10.1021/jp072183i) 17625809 10.1021/jp072183i

[68] Cable JR Tubergen MJ Levy DH 1987 Laser desorption molecular beam spectroscopy: the electronic spectra of tryptophan peptides in the gas phase. J. Am. Chem. Soc. 109, 6198–6199 (doi:10.1021/ja00254a057)

[69] Piuzzi F Dimicoli I Mons M Tardivel B Zhao Q 2000 A simple laser vaporization source for thermally fragile molecules coupled to a supersonic expansion: application to the spectroscopy of tryptophan. Chem. Phys. Lett. 320, 282–288 (doi:10.1016/s0009-2614(00)00242-6)

[70] Cohen R Brauer B Nir E Grace L de Vries MS 2000 Resonance-enhanced multiphoton ionization spectroscopy of dipeptides. J. Phys. Chem. A. 104, 6351–6355 (doi:10.1021/jp000413m)

[71] Hunig I Kleinermanns K 2004 Conformers of the peptides glycine-tryptophan, tryptophan-glycine and tryptophan-glycine-glycine as revealed by double resonance laser spectroscopy. Phys. Chem. Chem. Phys. 6, 2650–2658 (doi:10.1039/b316295h)

[72] Nir E Kleinermanns K Grace L de Vries MS 2001 On the photochemistry of purine nucleobases. J. Phys. Chem. A 105, 5106–5110 (doi:10.1021/jp0030645)

[73] Karas M Bachmann D Hillenkamp F 1985 Influence of the wavelength in high-irradiance ultraviolet laser desorption mass spectrometry of organic molecules. Anal. Chem. 57 (doi:10.1021/ac00291a042)

[74] Sunner J Dratz E Chen Y-C 1995 Graphite surface-assisted laser desorption/ionization time-of-flight mass spectrometry of peptides and proteins from liquid solutions. Anal. Chem. 67, 4335–4342 (doi:10.1021/ac00119a021) 8633776 10.1021/ac00119a021

[75] Dale MJ Knochenmuss R Zenobi R 1996 Graphite/liquid mixed matrices for laser desorption/ionization mass spectrometry. Anal. Chem. 68, 3321–3329 (doi:10.1021/ac960558i) 21619267 10.1021/ac960558i

[76] Continetti RE 2001 Coincidence spectroscopy. Annu. Rev. Phys. Chem. 52, 165–192 (doi:10.1146/annurev.physchem.52.1.165) 11326063 10.1146/annurev.physchem.52.1.165

[77] Zavriyev A Fischer I Villeneuve DM Stolow A 1995 Ponderomotive effects in zero kinetic energy photoelectron spectroscopy with intense femtosecond pulses. Chem. Phys. Lett. 234, 281–288 (doi:10.1016/0009-2614(95)00049-a)

[78] Smith VR Samoylova E Ritze HH Radloff W Schultz T 2010 Excimer states in microhydrated adenine clusters. Phys. Chem. Chem. Phys. 12, 9632–9636 (doi:10.1039/c003967e) 20556283 10.1039/c003967e

[79] Taherkhani M Riese M BenYezzar M Muller-Dethlefs K 2010 A novel experimental system of high stability and lifetime for the laser-desorption of biomolecules. Rev. Sci. Instrum. 81, 063101 (doi:10.1063/1.3373977) 20590219 10.1063/1.3373977

[80] Nir E Imhof P Kleinermanns K 2000 REMPI spectroscopy of laser desorbed guanosines. J. Am. Chem. Soc. 122, 8091–8092 (doi:10.1021/ja000502c) 10.1021/ja000502c 28876914

[81] Nir E Kleinermanns K de Vries MS 2000 Pairing of isolated nucleic-acid bases in the absence of the DNA backbone. Nature 408, 949–951 (doi:10.1038/35050053) 11140676 10.1038/35050053

[82] Li L Lubman DM 1989 Resonance enhanced multiphoton ionization of nucleosides by using pulsed-laser desorption in supersonic beam mass spectrometry. Int. J. Mass Spectrom. Ion Process. 88, 197–210 (doi:10.1016/0168-1176(89)85016-5)

[83] Gengeliczki Z 2010 Effect of substituents on the excited-state dynamics of the modified DNA bases 2,4-diaminopyrimidine and 2,6-diaminopurine. Phys. Chem. Chem. Phys. 12, 5375–5388 (doi:10.1039/b917852j) 20379571 10.1039/b917852j

[84] Nachtigallova D Lischka H Szymczak JJ Barbatti M Hobza P Gengeliczki Z Pino G Callahan MP de Vries MS 2010 The effect of C5 substitution on the photochemistry of uracil. Phys. Chem. Chem. Phys. 12, 4924–4933 (doi:10.1039/b925803p) 20445900 10.1039/b925803p

[85] Meijer G Vries MS Hunziker HE Wendt HR 1990 Laser desorption jet-cooling of organic molecules. Appl. Phys. B 51, 395–403 (doi:10.1007/bf00329101)

[86] Lindner B Seydel U 1985 Laser desorption mass spectrometry of nonvolatiles under shock wave conditions. Anal. Chem. 57, 895–899 (doi:10.1021/ac00281a027)

[87] Belshaw L Calegari F Duffy MJ Trabattoni A Poletto L Nisoli M Greenwood JB 2012 Observation of ultrafast charge migration in an amino acid. J. Phys. Chem. Lett. 3, 3751–3754 (doi:10.1021/jz3016028) 10.1021/jz3016028 26291106

[88] Shea RC Petzold CJ Campbell JL Li S Aaserud DJ Kenttamaa HI 2006 Characterization of laser-induced acoustic desorption coupled with a Fourier transform ion cyclotron resonance mass spectrometer. Anal. Chem. 78, 6133–6139 (doi:10.1021/ac0602827) 16944895 10.1021/ac0602827

[89] Calvert CR 2012 LIAD-fs scheme for studies of ultrafast laser interactions with gas phase biomolecules. Phys. Chem. Chem. Phys. 14, 6289–6297 (doi:10.1039/c2cp23840c) 22322861 10.1039/c2cp23840c

[90] Shea RC Habicht SC Vaughn WE Kenttamaa HI 2007 Design and characterization of a high-power laser-induced acoustic desorption probe coupled with a Fourier transform ion cyclotron resonance mass spectrometer. Anal. Chem. 79, 2688–2694 (doi:10.1021/ac061597p) 17319645 10.1021/ac061597p PMC2547414

[91] Becker D Adhikary A Sevilla MD 2007 Charge migration in DNA: physics, chemistry and biology perspectives, pp. 139–175 New York: Springer

[92] Weinkauf R Schanen P Metsala A Schlag EW Bürgle M Kessler H 1996 Highly efficient charge transfer in peptide cations in the gas phase: threshold effects and mechanism. J. Phys. Chem. 100, 18 (doi:10.1021/jp960926m)

[93] Bald I Dabkowska I Illenberger E 2008 Probing biomolecules by laser-induced acoustic desorption: electrons at near zero electron volts trigger sugar-phosphate cleavage. Angew. Chem. Int. Ed. Engl. 47, 8518–8520 (doi:10.1002/anie.200803382) 18825764 10.1002/anie.200803382

[94] Zinovev AV Veryovkin IV Moore JF Pellin MJ 2007 Laser-driven acoustic desorption of organic molecules from back-irradiated solid foils. Anal. Chem. 79, 8232–8241 (doi:10.1021/ac070584o) 17914890 10.1021/ac070584o

[95] Nyadong L McKenna AM Hendrickson CL Rodgers RP Marshall AG 2011 Atmospheric pressure laser-induced acoustic desorption chemical ionization Fourier transform ion cyclotron resonance mass spectrometry for the analysis of complex mixtures. Anal. Chem. 83, 1616–1623 (doi:10.1021/ac102543s) 21306132 10.1021/ac102543s

[96] Lodish H Berk A Zipursky SL Matsudaira P Baltimore D Darnell J 2000 Molecular cell biology New York, NY: W.H. Freeman

[97] Dole M 1968 Molecular beams of macroions. J. Chem. Phys. 49, 2240 (doi:10.1063/1.1670391)

[98] Yamashita M Fenn JB 1984 Negative ion production with the electrospray ion source. J. Phys. Chem. 88, 4671–4675 (doi:10.1021/j150664a046)

[99] Fenn JB 2003 Electrospray wings for molecular elephants (Nobel lecture). Angew. Chem. Int. Ed. 42, 3871–3894 (doi:10.1002/anie.200300605) 10.1002/anie.200300605 12949861

[100] Bakhtiar R Thomas JJ Siuzdak G 2000 Mass spectrometry in viral proteomics. Acc. Chem. Res. 33, 179–187 (doi:10.1021/ar9801200) 10727207 10.1021/ar9801200

[101] Nie Z Tzeng YK Chang HC Chiu CC Chang CY Chang CM Tao MH 2006 Microscopy-based mass measurement of a single whole virus in a cylindrical ion trap. Angew. Chem. Int. Ed. Engl. 45, 8131–8134 (doi:10.1002/anie.200603839) 17146754 10.1002/anie.200603839

[102] Cech NB Enke CG 2001 Practical implications of some recent studies in electrospray ionization fundamentals. Mass. Spectrom. Rev. 20, 362–387 (doi:10.1002/mas.10008) 11997944 10.1002/mas.10008

[103] Fujihara A Noguchi N Yamada Y Ishikawa H Fuke K 2009 Microsolvation and protonation effects on geometric and electronic structures of tryptophan and tryptophan-containing dipeptides. J. Phys. Chem. A 113, 8169–8175 (doi:10.1021/jp902451k) 19569705 10.1021/jp902451k

[104] Badu-Tawiah AK Campbell DI Cooks RG 2012 Reactions of microsolvated organic compounds at ambient surfaces: droplet velocity, charge state, and solvent effects. J. Am. Soc. Mass Spectrom. 23, 1077–1084 (doi:10.1007/s13361-012-0365-3) 22427191 10.1007/s13361-012-0365-3

[105] Kang H 2005 Ultrafast deactivation mechanisms of protonated aromatic amino acids following UV excitation. Phys. Chem. Chem. Phys. 7, 394–398 (doi:10.1039/b414986f) 19785164 10.1039/b414986f

[106] Kang H 2005 Photoinduced processes in protonated tryptamine. J. Chem. Phys. 122, 084307 (doi:10.1063/1.1851503) 10.1063/1.1851503 15836039

[107] Gregoire G Kang H Dedonder-Lardeux C Jouvet C Desfrancois C Onidas D Lepere V Fayeton JA 2006 Statistical versus non-statistical deactivation pathways in the UV photo-fragmentation of protonated tryptophan-leucine dipeptide. Phys. Chem. Chem. Phys. 8 (doi:10.1039/b510406h) 10.1039/b510406h 16482251

[108] Nolting D Weinkauf R Hertel IV Schultz T 2007 Excited-state relaxation of protonated adenine. Chem. Phys. Chem. 8, 751–755 (doi:10.1002/cphc.200600727) 17366508 10.1002/cphc.200600727

[109] Chatterley AS Johns AS Stavros VG Verlet JR 2013 Base-specific ionization of deprotonated nucleotides by resonance enhanced two-photon detachment. J. Phys. Chem. A 117, 5299–5305 (doi:10.1021/jp4041315) 23642262 10.1021/jp4041315

[110] Kang H Dedonder-Lardeux C Jouvet C Gregoire G Desfrancois C Schermann JP Barat M Fayeton JA 2005 Control of bond-cleaving reactions of free protonated tryptophan ion by femtosecond laser pulses. J. Phys. Chem. A 109, 2417–2420 (doi:10.1021/jp0407167) 16833540 10.1021/jp0407167

[111] Nolting D Schultz T Hertel IV Weinkauf R 2006 Excited state dynamics and fragmentation channels of the protonated dipeptide H2N-Leu-Trp-COOH. Phys. Chem. Chem. Phys. 8, 5247–5254 (doi:10.1039/b609726j) 17203149 10.1039/b609726j

[112] Vonderach M Ehrler OT Weis P Kappes MM 2011 Combining ion mobility spectrometry, mass spectrometry, and photoelectron spectroscopy in a high-transmission instrument. Anal. Chem. 83, 1108–1115 (doi:10.1021/ac1029677) 21214198 10.1021/ac1029677

[113] Vonderach M Ehrler OT Matheis K Weis P Kappes MM 2012 Isomer-selected photoelectron spectroscopy of isolated DNA oligonucleotides: phosphate and nucleobase deprotonation at high negative charge states. J. Am. Chem. Soc. 134, 7830–7841 (doi:10.1021/ja300619j) 22524691 10.1021/ja300619j

[114] Kim MH Shen L Tao H Martinez TJ Suits AG 2007 Conformationally controlled chemistry: excited-state dynamics dictate ground-state reaction. Science 315, 1561–1565 (doi:10.1126/science.1136453) 17363670 10.1126/science.1136453

[115] Kosma K Schroter C Samoylova E Hertel IV Schultz T 2009 Excited-state dynamics of cytosine tautomers. J. Am. Chem. Soc. 131, 16 (doi:10.1021/ja907355a) 19874018 10.1021/ja907355a

[116] Lecointre J Roberts GM Horke DA Verlet JR 2010 Ultrafast relaxation dynamics observed through time-resolved photoelectron angular distributions. J. Phys. Chem. A 114, 11 (doi:10.1021/jp1028855) 10.1021/jp1028855 20961158

[117] Yang X Wang XB Vorpagel ER Wang LS 2004 Direct experimental observation of the low ionization potentials of guanine in free oligonucleotides by using photoelectron spectroscopy. Proc. Natl Acad. Sci. USA 101, 17 (doi:10.1073/pnas.0405157101) 10.1073/pnas.0405157101 PMC539719 15591345

[118] Horke DA Chatterley AS Verlet JRR 2012 Femtosecond photoelectron imaging of aligned polyanions: probing molecular dynamics through the electron–anion coulomb repulsion. J. Phys. Chem. Lett. 3, 834–838 (doi:10.1021/jz3000933) 10.1021/jz3000933 26286406

[119] Mercier SR Boyarkin OV Kamariotis A Guglielmi M Tavernelli I Cascella M Rothlisberger U Rizzo TR 2006 Microsolvation effects on the excited-state dynamics of protonated tryptophan. J. Am. Chem. Soc. 128, 16 (doi:10.1021/ja065980n) 17177445 10.1021/ja065980n

[120] Verlet JRR 2008 Femtosecond spectroscopy of cluster anions: insights into condensed-phase phenomena from the gas-phase. Chem. Soc. Rev. 37, 505–517 (doi:10.1039/b700528h) 18224260 10.1039/b700528h

[121] Harris SJ 2013 Comparing molecular photofragmentation dynamics in the gas and liquid phases. Phys. Chem. Chem. Phys. 15, 6567–6582 (doi:10.1039/c3cp50756d) 23552482 10.1039/c3cp50756d

